# Effect of standardized early weight-bearing training on postoperative rehabilitation in older adults with intertrochanteric femoral fractures: a randomized controlled trial

**DOI:** 10.1186/s12877-026-07533-4

**Published:** 2026-04-22

**Authors:** Yue Ma, Yan Fang, Yan Zhao, Hui Shi, Li Ding, Jingjuan Liang

**Affiliations:** 1 Department of nursing, Huashan Hospital, Fudan University, 12 Middle Urumqi Road, Shanghai, 200040 P. R. China; 2 Department of Rehabilitation, Huashan Hospital, Fudan University, Shanghai, 200040 P. R. China

**Keywords:** Older adults, Early weight-bearing, Intertrochanteric femoral fracture, Rehabilitation

## Abstract

**Aims:**

To evaluate the impact of standardized early weight-bearing training on postoperative rehabilitation in older adults with intertrochanteric femoral fractures.

**Methods:**

A total of 54 older adults with intertrochanteric femoral fractures admitted to the Department of Orthopedics, Huashan Hospital, Fudan University, between October 2023 and April 2025 were randomly allocated to a control group (*n* = 27) or an intervention group (*n* = 27). The control group received routine orthopedic rehabilitation, with weight-bearing initiated 4 weeks postoperatively, whereas the intervention group additionally received a standardized early weight-bearing program beginning 2–3 days after surgery. Outcomes including hip function, pain, quality of life, exercise adherence, Functional Independence Measure (FIM) scores, and weight-bearing duration were assessed at baseline, one day before discharge, and one month after discharge.

**Results:**

Baseline characteristics did not differ significantly between the two groups. Compared with the control group, the intervention group demonstrated significantly greater improvement in hip function, with higher Harris Hip Scores at one day before discharge (Z = − 2.506, P_FDR_= 0.018, *r* = 0.34) and at one month after discharge (Z = − 3.239, P_FDR_= 0.003, *r* = 0.44), linear mixed-effects modeling revealed a significant group-by-time interaction (β = 6.34, 95% CI: 2.88–9.80; *P* < 0.001), with the between-group difference reaching approximately 9.6 points at follow-up. Resting pain scores were significantly lower in the intervention group at pre-discharge (Z = − 2.770, P_FDR_ = 0.010) and one month after discharge (Z = − 2.326, P_FDR_= 0.024), with a significant group-by-time interaction indicating greater early pain relief. At follow-up, the intervention group achieved higher FIM scores, including the motor subscale (Hedges’ g = − 0.892), cognitive subscale (Hedges’ g = − 1.097), and total score (Hedges’ g = − 0.993). Weight-bearing duration was significantly longer in the intervention group (15.52 ± 8.02 vs. 10.59 ± 7.86 min; P_FDR_ = 0.027). The intervention group demonstrated significantly higher scores in the psychological domain of quality of life (*P* < 0.05).

**Conclusion:**

Standardized early weight-bearing training was associated with improved hip function, pain relief, functional independence, exercise compliance, psychological well-being, and weight-bearing performance in older adults with intertrochanteric femoral fractures.

**Trial registration:**

The trial was registered at the Chinese Clinical Trials Registry; retrospectively registered; registration number: ChiCTR2500095906; date of registration:2025/01/15.

**Supplementary Information:**

The online version contains supplementary material available at 10.1186/s12877-026-07533-4.

## Background

With the intensification of global population aging, the incidence of fractures among older adults has steadily increasing [1]. Hip fractures have become one of the top ten causes of disability and reduced life expectancy in individuals aged 65 years and older [2]. According to estimates from the International Osteoporosis Foundation, by 2050 there will be approximately six million hip fracture cases worldwide each year, of which nearly 90% will occur in patients over the age of 65, with intertrochanteric femoral fractures accounting for about 50% [3]. In clinical practice, most older adults with intertrochanteric femoral fractures undergo functional rehabilitation exercises in a bed-rest setting, which often results in delayed weight-bearing and a lack of standardized protocols. According to the 2023 Chinese Expert Consensus on Postoperative Weight-Bearing for Lower Limb Fractures, patients who pass safety assessments may begin early, tolerable weight-bearing rehabilitation under close monitoring [4]. This gap between evidence and practice stems from a combination of system-level constraints (e.g., limited access to early physiotherapy, high nurse-to-patient ratios, and fragmented postoperative care pathways) and deeply ingrained clinical beliefs, such as concerns about implant failure in osteoporotic bone or the perception that frail older adults are “too weak” to mobilize safely in the immediate postoperative period. Against this backdrop, this study aimed to develop and implement a standardized early weight-bearing training protocol for older adults after intertrochanteric femoral fracture surgery, with the goal of enhancing treatment confidence, improving quality of life, and evaluating its impact on postoperative recovery.

## Materials and methods

### Participants and grouping

This study was approved by the Huashan Hospital, Fudan University, China (approval number: Approval No. 2023临审第[817]号, approval date: 2023/10/18). Letters of informed consent signed by patients were obtained. This study was conducted in accordance with the principles of the Declaration of Helsinki. The trial was registered at the Chinese Clinical Trial Registry (registration number: ChiCTR2500095906; date of registration:2025/01/15). A total of 54 older adults with intertrochanteric femoral fractures who were admitted to the Department of Orthopedics, Huashan Hospital, Fudan University, between October 2023 and April 2025 were enrolled in this study.

**Fig. 1:**
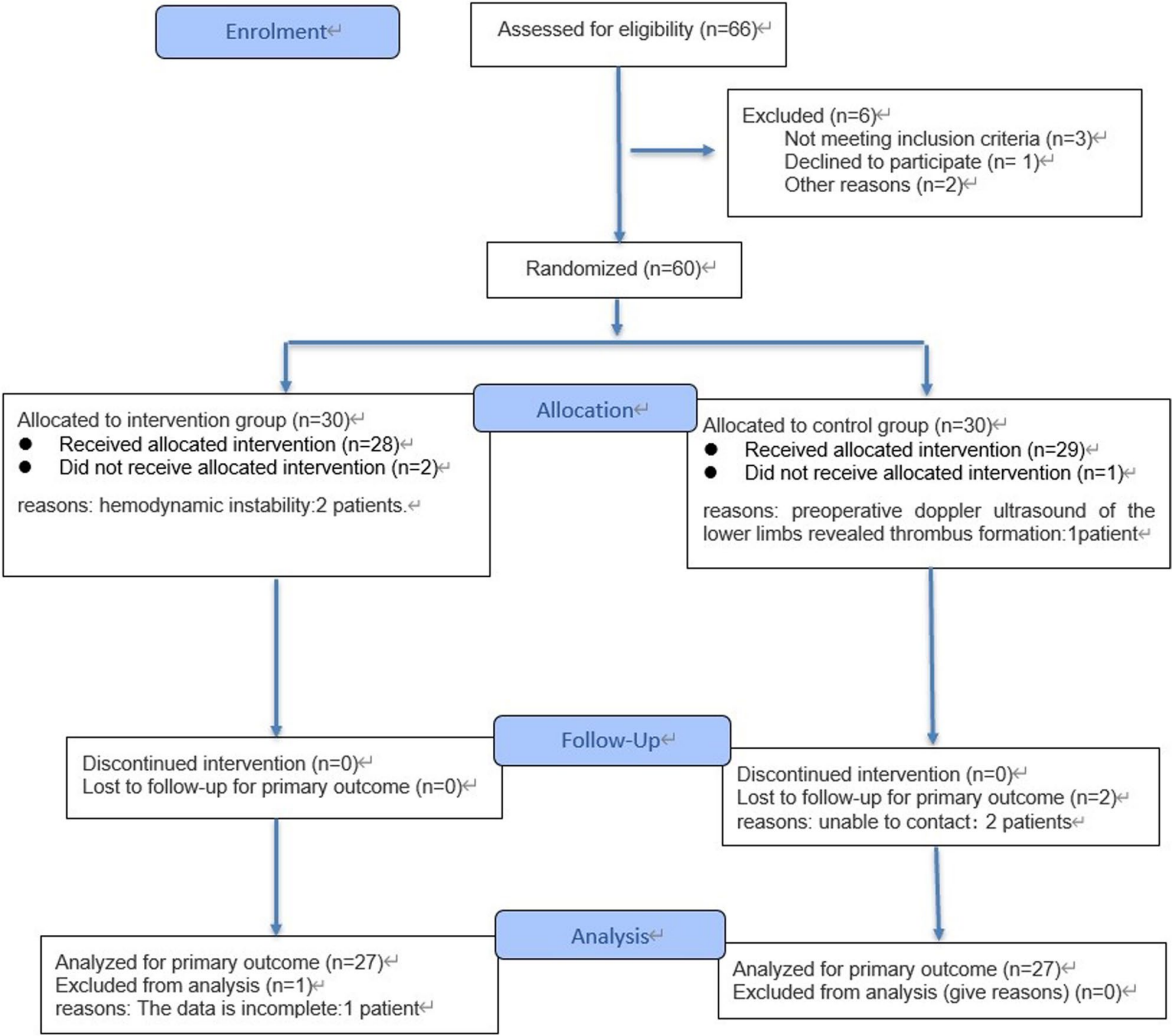
Flowchart according to CONSORT statement for the report of randomized trials

Participant Flow: a total of 66 potential patients were assessed for eligibility. 6 patients were excluded due to not meeting inclusion criteria or declining to participate. The remaining 60 patients were randomized to the intervention group (*n* = 30) and the control group (*n* = 30). In the intervention group, 2 patients withdrew due to postoperative hemodynamic instability; in the control group, 1 patient withdrew due to preoperative doppler ultrasound of the lower limbs revealed thrombus formation. During follow-up, 2 patients in the control group were lost to follow-up, and 1 patient in the intervention group had severely missing data. Ultimately, 27 patients in each group were included in the final analysis. All enrolled patients were analyzed for the primary outcome (Fig. 1).

### Inclusion and exclusion criteria

Inclusion criteria:

1. Patients with a confirmed diagnosis of intertrochanteric femoral fracture;
2. Age ≥ 65 years;
3. Fractures caused by low-energy trauma;
4. Patients undergoing surgical treatment with closed reduction and intramedullary fixation using Proximal Femoral Nail Antirotation (PFNA——Synthes, Switzerland).

Exclusion criteria:

1. Pathological intertrochanteric femoral fractures;
2. Patients with Evans type II fractures (Original version classification in 1949);
3. Old intertrochanteric fractures or revision cases due to nonunion;
4. Patients who had lost weight-bearing or ambulatory ability prior to fracture;
5. Patients with multiple fractures or polytrauma;
6. Patients with severe osteoporosis;
7. Patients who were deemed unlikely to complete the postoperative rehabilitation program and follow-up schedule: such as Mini-Mental State Examination (MMSE)<21 points.

Study discontinuation criteria:

1. Patients in critical condition;
2. Patients who withdrew from the study midway;
3. Patients lost to follow-up.

### Study protocol

#### Establishment of a multidisciplinary clinical-rehabilitation-nursing team

A 10-member multidisciplinary team was established in the orthopedic ward of Huashan Hospital, Fudan University, Shanghai, including 1 chief physician, 1 attending physician, 1 deputy chief nurse, 2 senior nurses, 4 registered nurses, and 1 rehabilitation therapist.

Inclusion criteria for team members were:

1. At least three years of professional experience;
2. Proficiency in the use of common clinical assessment scales and specialized knowledge of the relevant diseases;
3. Bachelor’s degree or higher;
4. Strong communication and interpersonal skills.

All team members received standardized training and assessment in disease knowledge and rehabilitation protocols prior to participation in the study.

### Standardized surgical protocol and radiographic stability criteria

All patients underwent PFNA fixation following standardized institutional operative protocols to ensure procedural consistency and group comparability. All procedures were performed by the same orthopedic trauma team, including three attending surgeons with > 10 years of experience, using the same surgical instruments and fluoroscopy equipment. Intraoperative complications were documented in operative notes and monitored during follow-up. Postoperative radiographs were obtained at defined intervals to verify the maintenance of reduction and implant integrity. Only cases demonstrating radiographic stability were permitted to initiate or progress early weight-bearing training.

### Control group

Patients in the control group received routine orthopedic rehabilitation care. Preoperatively, they were instructed in muscle-strengthening exercises (quadriceps isometric contractions, bridge exercises), joint mobility exercises (ankle dorsiflexion and plantarflexion, hip and knee range-of-motion exercises), and positioning management (elevation of the affected limb with a soft pillow).

On postoperative day 1, patients were guided through bedside exercises:

1. Toe and ankle pump exercises: actively plantarflex and dorsiflex the foot for 5 s each;
2. Hip and knee flexion-extension exercises: sliding the heel along the bed while bending and extending the knee;
3. Straight leg raises: keep the affected limb straight and in a neutral position. Place only a soft pillow under the thigh. Lift the lower leg about 10 cm off the bed and hold for 5 to 10 s; if unable, move the limb horizontally outward and return to starting position;
4. Quadriceps isometric contractions: pressing the knee downward for 5 s while supine;
5. Supine bridge exercises: strengthening the lumbar and back muscles using a five-point support;
6. Sitting training: allowing both lower limbs to hang naturally for 15 min to experience weight bearing.

On postoperative days 2–3, the clinical-rehabilitation-nursing team provided written and verbal education on early weight-bearing exercises, which patients could also review via the hospital’s WeChat public account. Gradual weight-bearing was initiated four weeks postoperatively following physician assessment.

### Intervention group

Patients in the intervention group received all routine care provided to the control group, supplemented with a full-cycle clinical-rehabilitation-nursing management program based on the Enhanced Recovery After Surgery (ERAS) concept, aimed at promoting early weight-bearing. The intervention included the following components:

1. Early Weight-Bearing Initiation (Postoperative Days 2–3): After evaluation—including monitoring of vital signs, manual muscle testing (bilateral upper limbs ≥ grade 4, bilateral lower limbs ≥ grade 3), intraoperative assessment of fracture biomechanical stability and soft tissue condition, and screening for other discomforts such as dizziness, chest tightness, or fatigue—patients were assisted by the multidisciplinary team to sit at the bedside with legs hanging for 15 min. If no adverse symptoms occurred, early weight-bearing training was initiated. This began with bedside postural adaptation and center-of-gravity transfer training: patients were guided to place feet shoulder-width apart, stand with the body weight primarily on the unaffected limb, extend the affected limb onto a digital weighing scale, and gradually shift their weight onto the affected limb with the assistance of a walker.
2. Individualized Early Weight-Bearing Plan: During hospitalization, a personalized early weight-bearing plan was developed for each patient and dynamically adjusted based on their feedback. Patients were also provided with instructional videos through the hospital’s WeChat public account to reinforce training guidance.
3. Standardized Load Progression: Early weight-bearing load was objectively quantified using a digital scale. The initial load was set at 5–10% of body weight (or adjusted based on the patient‘s subjective feeling and feeling of pain). The load was progressively increased by approximately 5% of body weight per day or every other day, as tolerated. During each session, patients were guided to shift their weight onto the scale until the display reached the target load, each group held the position for 30–60 s, 2 sets of 3–5 reps per day.
4. Criteria for Program Modification: The load progression plan was dynamically adjusted based on daily performance. If a patient tolerated the current load well (NRS ≤ 2) for two consecutive days, the load was increased per the protocol. If a patient failed to tolerate the current target for three consecutive days due to pain or fear, a multidisciplinary review was triggered to reassess fracture stability, pain management, and psychological status, potentially leading to a more gradual plan or adjunctive interventions.

### Safety measures during intervention

Safety during early weight-bearing training was ensured through the following measures:

1. Pre-training Assessment: Prior to each session, physicians evaluated the patient’s overall and local condition. Overall assessment included mental status, consciousness, and sensory-motor function of the limbs, as well as willingness to cooperate. Local assessment focused on the surgical site, checking for bleeding or exudation.
2. Continuous Monitoring: Vital signs were continuously monitored during the intervention to ensure patient safety.
3. Post-training Radiographic Verification: After the final early weight-bearing session during hospitalization, radiographs were obtained to confirm the stability of the internal fixation at the fracture site.
4. Tolerance Thresholds and Stopping Criteria: The intervention continued only if the patient reported pain ≤ 3 on the Numeric Rating Scale (0–10) during the activity. The session was immediately paused if any of the following occurred: a sudden increase in hip pain (NRS increase ≥ 2), sharp or stabbing pain at the surgical site, onset of dizziness, chest tightness, shortness of breath, profuse sweating, fresh bleeding from the incision, or significant patient anxiety and refusal.

### Timing and intensity of rehabilitation exercises

1. Toe and Ankle Pump Exercises: Performed during daytime, 5–10 min every hour.
2. Hip and Knee Flexion-Extension, Straight Leg Raises, Quadriceps Isometric Contractions, Bridge Exercises: 2–3 sets per day, 5–10 repetitions per set.
3. Sitting Training: Twice daily.
4. Early Weight-Bearing Training: Two sets a day, each group 3–5 times, each time 30–60 s, digital weighing scale was used to measure the load on the affected limb, the clock recorded the duration of the load.

The intensity and duration of functional exercises were adjusted according to patient tolerance. Frequency could be increased as the patient’s capacity improved, provided that pain remained tolerable and no additional discomfort occurred.

### Follow-up

One month after discharge, patients were followed up via WeChat or telephone to assess adherence to rehabilitation exercises, quality of life, and early weight-bearing progress. All findings were recorded for subsequent analysis.

### Outcome measures

1. Harris Hip Score (HHS): The Harris Hip Score is one of the most widely used tools for evaluating hip joint function. It assesses pain, deformity, range of motion, function, gait, use of walking aids, and walking distance [5]. The maximum score is 100 points, with < 70 considered poor, 70–79 fair, 80–89 good, and 90–100 excellent. We took this scale as the primary outcome measure.
2. Numeric Rating Scale (NRS) for Pain: Pain intensity was assessed using an 11-point scale ranging from 0 to 10, where 0 indicates no pain and 10 indicates the worst imaginable pain. Scores were categorized as follows: 0 = no pain, 1–3 = mild pain, 4–6 = moderate pain, and 7–10 = severe pain. The NRS has demonstrated good reliability and validity and is recommended by the American Geriatrics Society as the preferred tool for pain assessment in older adults [6].
3. World Health Organization Quality of Life Assessment Brief Form (WHOQOL-BREF): This is a simplified version of the WHO Quality of Life assessment, consisting of 26 items across four domains: physical, psychological, social, and environmental. Total scores range from 26 to 130, with higher scores indicating better quality of life. The scale has a Cronbach’s alpha of 0.798, indicating good internal consistency [7].
4. Orthopedic Exercise Adherence Scale: This scale consists of 15 items across three dimensions: physical, psychological, and active learning adherence. A 5-point Likert scale is used, with a total score ranging from 15 to 75. Scores ≤ 20 indicate low adherence, 21–54 indicate partial adherence, and ≥ 55 indicate high adherence. The scale demonstrates good psychometric properties, with a Cronbach’s alpha of 0.930 and content validity index of 0.936, and was used with permission from the original author [8].
5. Functional Independence Measure (FIM): The FIM was used to assess activities of daily living, including 13 motor items and 5 cognitive items. The total score ranges from 18 to 126, with higher scores indicating greater levels of functional independence. The scale demonstrates good reliability [9].
6. Weight-Bearing Data Recording: The duration of weight-bearing on the affected limb was recorded at the last follow-up one month after discharge in both the intervention and control groups.

### Data collection

1. On the day of admission, general demographic and clinical data were collected, including age, sex, length of hospital stay, education level, marital status, history of alcohol consumption and smoking, and presence of chronic diseases. Baseline assessments included quality of life, Harris Hip Score, pain (both at rest and during activity), and the Functional Independence Measure.
2. On the day before discharge, pain, Harris Hip Score, and the Orthopedic Exercise Adherence Scale were recorded. During hospitalization, data were collected by trained nurses, with the head nurse conducting unscheduled supervision to ensure the quality of rehabilitation training.
3. At one-month follow-up after discharge, quality of life, Harris Hip Score, pain (resting and activity-related), Orthopedic Exercise Adherence Scale, Functional Independence Measure, and the final weight-bearing duration of the affected limb were collected. Harris Hip Scores were obtained during outpatient visits, the last weight-bearing duration was reported by caregivers, and all other scales were collected through an online platform.

### Statistical analyses

#### Sample-size analysis

The Harris Hip Score was selected as the primary outcome measure. The sample size was calculated a priori using PASS 2021 software for a two-sample, two-sided t-test comparing means. Based on data from a previous study [10], the mean postoperative Harris scores of the intervention and control groups were assumed to be 82.84 ± 9.55 and 72.51 ± 10.44. This yielded an expected mean difference of 10.33 points. Assuming unequal variances between groups, with a two-sided significance level of α = 0.05 and a statistical power of 1 – β = 0.90, the initial calculation indicated a requirement of 21 participants per group. Allowing for a 20% dropout rate, 27 patients were needed in each group.

### Randomization and blinding

Randomization was performed by an independent statistician using block randomization with a block size of 4, implemented in Microsoft Excel. A total of 14 blocks were generated: the first 13 blocks each contained 4 allocation sequences, and the final block contained 2 sequences to accommodate the total sample size of 54 participants. Within each block, participants were randomly assigned in a 1:1 ratio to either the intervention or control group, ensuring balanced allocation (2:2 in full blocks and 1:1 in the final partial block). The assignment information was sealed in opaque, sequentially numbered envelopes by the independent statistician. These envelopes were kept securely by a research coordinator in the ward who was not involved in participant assessment or intervention delivery. Once patients met the inclusion criteria and signed informed consent, the corresponding envelope was opened in the presence of the patient or the principal investigator, strictly in sequential order of enrollment. Ultimately, 27 patients were assigned to the intervention group and 27 to the control group. During the trial, a single-blind protocol was maintained for outcome assessors; specifically, the nurse responsible for all follow-up procedures and data collection remained blinded to the treatment allocations throughout the study period to minimize ascertainment bias. To maintain blinding: (1) The outcome assessor was an orthopedic specialist nurse not involved in the daily ward care or rehabilitation training delivery. (2) All on-site functional assessments (e.g., Harris Hip Score) were conducted in a separate outpatient assessment room, not in the ward. (3) Patients and caregivers were instructed not to disclose details of their rehabilitation regimen to the assessor. The assessor documented perceived group allocation after each assessment; no unblinding was reported for any patient. While the intervention group received early weight-bearing training and the control group received standard care, the enrollment process, follow-up procedures, and assessment methods were identical for both groups, ensuring procedural similarity between the interventions.

### Data analysis

Data analyses were performed using SPSS version 27.0. Continuous variables were expressed as mean ± standard deviation (x̄ ± s). For variables not normally distributed, data were presented as median and interquartile range (M, IQR). After testing for normality and homogeneity of variance, between-group comparisons were conducted using independent-sample t tests, and within-group comparisons were performed using paired-sample t tests. When assumptions were not met, nonparametric methods were applied. Categorical variables were expressed as frequencies (*n*). For missing measurements at specific time points, the last observation carried forward method was used to impute missing values. As a sensitivity analysis for the primary outcome (Harris Hip Score), a linear mixed-effects model (LMM) was employed. The model included random intercepts for participants and fixed effects for group, time, and their interaction term. This analytical approach accommodates missing data under the missing-at-random (MAR) assumption without the need for imputation. To control for multiple comparisons, *P* values were adjusted using the Benjamini–Hochberg procedure to control the false discovery rate (FDR) at q = 0.05.

### Quality control

#### Pre-intervention preparation

1. Feasibility Assessment: The intervention protocol was reviewed and revised with input from clinical trauma surgeons and nursing experts.
2. Eligibility Criteria Management: Strict inclusion and exclusion criteria were established, and no arbitrary modification or withdrawal of cases was permitted throughout the study.

### Intervention implementation

1. Data Quality Control: (i) scales were distributed only to participants meeting the inclusion criteria; (ii) invalid scales were excluded after review by the investigators, and only valid responses were included in the analysis.
2. Individualized Intervention Adjustment: (i) Professional evaluation and guidance were provided based on the patient’s condition and rehabilitation progress; (ii) rehabilitation training content was adjusted in a timely manner according to patient needs.

### Data collection

1. Standardized Training: All staff responsible for inpatient data collection underwent standardized training.
2. Scales Validity Assurance: Responses were checked immediately, errors corrected, and accuracy confirmed to ensure data validity.

## Results

Prior to selecting statistical tests, normality of all continuous outcome variables was assessed using the Shapiro-Wilk test and visual inspection of Q-Q plots; homogeneity of variance was evaluated using Levene’s test. Variables violating normality assumptions (including Harris Hip Score, pain scores) were analyzed using non-parametric methods (Table 1).

**Table 1:** Results of Shapiro-Wilk normality tests for outcome measures at each time point

Outcome Measure	Time Point	W Statistic	*p*-value	Normally Distributed?
Physical	Before intervention	0.976	0.359	Yes
	1 month	0.967	0.137	Yes
Psychological	Before intervention	0.978	0.407	Yes
	1 month	0.967	0.147	Yes
Social	Before intervention	0.960	0.068	Yes
	1 month	0.962	0.088	Yes
Environmental	Before intervention	0.960	0.068	Yes
	1 month	0.983	0.619	Yes
Harris Hip Scores	Before intervention	0.881	<0.001	No
	1 day before discharge	0.910	<0.001	No
	1 month after discharge	0.940	0.009	No
Orthopedic Exercise Compliance Scale Scores	1 day before discharge	0.978	0.429	Yes
	1 month after discharge	0.962	0.084	Yes
Resting pain	Pre-intervention	0.918	0.001	No
	One day before discharge	0.862	<0.001	No
	One month after discharge	0.329	<0.001	No
Activity-related pain	Pre-intervention	0.923	0.002	No
	One day before discharge	0.916	0.001	No
	One month after discharge	0.775	<0.001	No
FIM Motor subscale	Before Intervention	0.972	0.233	Yes
	1 Month After Discharge	0.963	0.092	Yes
FIM Cognitive subscale	Before Intervention	0.968	0.159	Yes
	1 Month After Discharge	0.962	0.087	Yes
FIM Total score	Before Intervention	0.980	0.514	Yes
	1 Month After Discharge	0.958	0.058	Yes
Weight-bearing duration	1 Month After Discharge	0.972	0.235	Yes

### The comparison of the patient characteristics between two groups

The baseline characteristics of the patients are presented in Table 2. A total of 54 patients were enrolled, with 27 in the control group and 27 in the intervention group. There were no statistically significant differences between the two groups in terms of age, length of hospital stay, sex, educational level, marital status, comorbidities, alcohol consumption, or smoking history (all *P* > 0.05). This indicates that the two groups were comparable at baseline.

**Table 2:** Baseline characteristics of patients in the control and intervention groups

Variable	Control group (*n* = 27)	Intervention group (*n* = 27)	χ²/Z	*P*-value
Age (years, mean ± SD)	79.7 ± 9.85	80.96 ± 8.65	-0.286	0.775
Length of stay (days, mean ± SD)	7.15 ± 2.49	6.33 ± 1.79	-1.046	0.295
Sex (*n*, %)			1.374	0.241
Male	11 (40.7%)	6 (22.2%)		
Female	16 (59.3%)	21 (77.8%)		
Educational level (*n*, %)			6.014	0.311
Bachelor’s degree or above	7 (25.9%)	1 (3.7%)		
Junior college	4 (14.8%)	3 (11.1%)		
High school	5 (18.5%)	7 (25.9%)		
Middle school	2 (7.4%)	3 (11.1%)		
Primary school	6 (22.2%)	8 (29.6%)		
Illiterate	3 (11.1%)	5 (18.5%)		
Marital status (*n*, %)			0.964	0.326
Married	23 (85.2%)	19 (70.4%)		
Unmarried/divorced/widowed	4 (14.8%)	8 (29.6%)		
Comorbidities (*n*, %)			1.321	0.250
Yes	25 (92.6%)	21 (77.8%)		
No	2 (7.4%)	6 (22.2%)		
Alcohol history (*n*, %)			0.101	0.750
Yes	6 (22.2%)	7 (25.9%)		
No	21 (77.8%)	20 (74.1%)		
Smoking history (*n*, %)			0.114	0.735
Yes	6 (22.2%)	5 (18.5%)		
No	21 (77.8%)	22 (81.5%)		

### Comparison of Harris Hip Scores between the two groups before and after intervention

Given the non-normal distribution of HHS, nonparametric tests and a linear mixed-effects model were used to assess between-group differences and repeated measurements across three time points, with the control group as the reference.

Between-group comparisons revealed significant differences in Harris Hip Scores at one day before discharge (Z = − 2.506, P_FDR_=0.018, *r* = 0.34) and one month after discharge (Z = − 3.239, P_FDR_=0.003, *r* = 0.44), indicating medium to moderate effect sizes. No significant difference was observed at baseline (Z = − 1.037, P_FDR_ = 0.225, *r* = 0.14), consistent with similar initial functional levels between groups. These findings suggest that the intervention led to progressively greater improvements in hip function over time (Table 3).

**Table 3:** Harris Hip Scores before and after intervention (M, IQR)

Time point	Control group (*n* = 27)	Intervention group (*n* = 27)	Z-value	*P*_FDR_-value
Before intervention	12 (24)	13 (10)	-1.037	0.225
1 day before discharge	35 (11)^ac^	42 (17)^ac^	-2.506	0.018
1 month after discharge	64 (15)^ad^	72 (16)^ad^	-3.239	0.003

a: between-group comparison, *P* < 0.05

c: within-group comparison vs. baseline, *P* < 0.05

d: within-group comparison vs. 1 day before discharge, *P* < 0.05

Linear mixed-effects modeling showed a significant Group × Time interaction (β = 6.34, 95% CI: 2.88 to 9.80; *P* < 0.001), indicating greater improvement in Harris Hip Score over time in the intervention group versus control. The between-group difference increased progressively, reaching an estimated 9.6 points at follow-up—exceeding the minimal clinically important difference (MCID) of 8–10 points. Baseline scores did not differ significantly (*P* = 0.209) (Supplementary Table 1).

### Comparison of Pain Scores between the two groups before and after intervention

A nonparametric test was used to compare pain score differences between the two groups before and after the intervention. A generalized estimating equation (GEE) model was employed to analyze repeated measures of resting pain and activity-related scores across three time points (admission, pre-discharge, and follow-up), Group 1 served as the reference (control group), and Group 2 represented the intervention group.

At admission, there was no significant difference in resting pain between the intervention and control groups. However, at one day before discharge (U = 216.50, Z = − 2.770, P_FDR_=0.010, *r* = 0.38) and one month after discharge (U = 297.00, Z = − 2.326, P_FDR_=0.024, *r* = 0.32), the intervention group reported significantly lower resting pain scores with medium effect sizes. No meaningful difference in activity-related pain was observed between groups at admission (U = 394.00, Z = 0.526, P_FDR_=0.422, *r* = 0.07). By one day before discharge, however, the intervention group demonstrated significantly lower pain scores (U = 229.50, Z = − 2.422, P_FDR_=0.02), reflecting a small-to-medium effect (*r* = 0.33). This improvement did not persist at the one-month follow-up (U = 324.50, Z = − 0.764, P_FDR_= 0.296, *r* = 0.10) (Table 4).

**Table 4:** Comparison of pain scale scores between the two groups (M, IQR)

Pain Type	Control Group (*n* = 27)			Intervention Group (*n* = 27)		
	Pre-intervention	One day before discharge	One month after discharge	Pre-intervention	One day before discharge	One month after discharge
Resting pain	3 (2)	1 (1)ᵃ	0 (0)ᵃ	3 (2)	1 (1)ᵃ	0 (0)ᵃ
Activity-related pain	5 (1)	4 (2)ᵃ	1 (1)	5 (3)	3 (2)ᵃ	1 (1)

a: between-group comparison, *P* < 0.05

GEE analysis showed that the intervention group had consistently lower resting pain than the control group over time (group effect: β = − 0.185, *P* = 0.013). Pain decreased significantly in both groups from admission to follow-up (time effect: *P* < 0.001). A significant group-by-time interaction at pre-discharge (β = − 0.519, *P* = 0.016) indicated greater early pain relief in the intervention group, with no baseline difference at admission (*P* = 0.662). (Supplementary Table 2).

Generalized estimating equation (GEE) analysis of activity-related pain scores showed that: the main effect of group was not statistically significant (β = − 0.148, 95% CI [–0.496 to 0.200], *P* = 0.404), indicating no overall difference between groups across the observation period. The main effect of time was highly significant (admission vs. follow-up: β = 4.778, *P* < 0.001; pre-discharge vs. follow-up: β = 2.407, *P* < 0.001), reflecting substantial pain reduction over time in both groups. A group-by-time interaction approached significance at pre-discharge, with the intervention group reporting 0.704 points lower activity pain than the control group (β = − 0.704, 95% CI [–1.419 to − 0.012], *P* = 0.054), suggesting a potential early benefit of the intervention. Collectively, while no significant overall group difference was observed, the intervention may facilitate earlier relief of activity-related pain before discharge (Supplementary Table 3).

### Comparison of quality of life scores between the two groups before and after intervention

Independent-sample t tests were used to compare the quality of life (QoL) scores in each domain between the two groups. Before the intervention, there were no statistically significant differences in any QoL domains between the groups. At one-month follow-up after discharge, scores in the physical, psychological, and environmental domains significantly improved compared with baseline in both groups (*P*<0.05). At one-month follow-up, between-group comparisons revealed that the intervention group reported significantly higher scores in the psychological domain of quality of life compared to the control group (t = − 2.304, P_FDR_=0.027, 95%CI [-1.154, -0.076]), with a medium effect size (Hedges’ g = -0.618). These findings indicate that standardized early weight-bearing training may exert its most meaningful influence on patients’ psychological well-being during early recovery (Table 5).

**Table 5:** Comparison of quality of life and functional independence measure scores between groups before and one month after discharge (x̄ ± s)

Outcome measures	Control Group (*n* = 27)		Intervention Group (*n* = 27)		t-value	*P*_FDR_-value
	Before Intervention	1 Month After Discharge	Before Intervention	1 Month After Discharge		
FIM Scale						
Motor subscale	36.26 ± 6.36	77.07 ± 16.99^ab^	34.78 ± 7.10	92.37 ± 16.81^ab^	-3.326	0.004
Cognitive subscale	23.63 ± 2.79	25.96 ± 3.30^ab^	24.67 ± 3.87	29.81 ± 3.62^ab^	-4.089	0.003
Total score	59.89 ± 7.45	103.33 ± 19.00^ab^	59.44 ± 9.09	122.19 ± 18.94^ab^	-3.702	0.003
Quality of Life						
Physical	11.47 ± 2.36	12.68 ± 2.40^b^	11.85 ± 2.71	13.59 ± 2.56^b^	-1.346	0.169
Psychological	10.21 ± 1.45	10.42 ± 2.52^ab^	9.77 ± 1.96	12.20 ± 3.12^ab^	-2.304	0.027
Social	11.46 ± 1.93	11.58 ± 1.42	11.11 ± 2.22	11.94 ± 1.85	-0.805	0.340
Environmental	13.24 ± 2.02	14.09 ± 2.11^b^	12.91 ± 2.88	14.65 ± 2.59^b^	-0.864	0.335

a: between-group comparison, *P* < 0.05

b: within-group comparison, *P* < 0.05

### Comparison of Functional Independence Measure (FIM) scores before and after intervention between the two groups

There was no significant difference in FIM scores between the two groups before the intervention. At one month after discharge, the intervention group demonstrated significantly higher FIM scores compared to the control group across all domains: motor subscale (t = − 3.326, P_FDR_=0.004, 95%CI[-1.441, -0.335], Hedges’ g = -0.892), cognitive subscale (t = − 4.089, P_FDR_=0.003, 95%CI[-1.658, 0.526], Hedges’ g = -1.097), and total score (t = − 3.702, P_FDR_=0.003, 95%CI[-1.548, 0.430], Hedges’ g= -0.993). These large to moderate effect sizes suggest that the intervention substantially improved both physical and cognitive functional independence in patients following hip surgery (Table 5).

### Comparison of orthopedic exercise compliance scale scores between the two groups

An independent samples t-test was conducted for comparative analysis. At one day before discharge, the intervention group showed significantly higher orthopedic exercise compliance scores than the control group (t = − 3.307, P_FDR_=0.004, 95%CI[-1.436, -0.331], Hedges’ g = -0.887), indicating a moderate to large effect size. This difference was further amplified at 1 month after discharge (t = − 3.714, P_FDR_=0.003, Hedges’ g=-0.996, 95%CI[-1.551, -0.433]), suggesting that the intervention effectively promoted long-term adherence to rehabilitation exercises (Table 6).

**Table 6:** Comparison of orthopedic exercise compliance scale scores between the two groups (x̄ ± s)

Time Point	Control Group (*n* = 27)	Intervention Group (*n* = 27)	t-value	*P*_FDR_-value
1 day before discharge	34.56 ± 10.44ᵃ	43.93 ± 10.38ᵃ	-3.307	0.004
1 month after discharge	37.78 ± 11.22ᵃᵇ	47.74 ± 8.28ᵃᵇ	-3.714	0.003

a: between-group comparison, *P* < 0.05

b: within-group comparison, *P* < 0.05

### Comparison of post-discharge weight-bearing performance between the two groups

Parametric analysis using an independent samples t-test revealed that the intervention group had a significantly longer weight-bearing duration (15.52 ± 8.02 min) compared to the control group (10.59 ± 7.86 min) at the same time point (t = − 2.280, P_FDR_=0.027, 95%CI [-1.147, -0.070], Hedges’ g = -0.611), indicating a moderate effect size. This suggests that the standardized early weight-bearing protocol effectively increased patients’ actual weight-bearing time during rehabilitation (Table 7).

**Table 7:** Comparison of weight-bearing duration between the two groups (x̄ ± s)

Weight-Bearing Data	Control Group (*n* = 27)	Intervention Group (*n* = 27)	t-value	*P*_FDR_-value
Duration (min)	10.59 ± 7.86	15.52 ± 8.02^a^	-2.280	0.027

a: between-group comparison, *P* < 0.05

## Discussion

The present study demonstrated that, following the intervention, both groups exhibited improvements in the physical, psychological, and environmental domains of quality of life, whereas no significant change was observed in the social domain. Notably, a significant between-group difference was detected only in the psychological domain, indicating that early weight-bearing training may have a particularly beneficial effect on psychological well-being. These findings suggest that implementing early weight-bearing rehabilitation can contribute to overall quality of life, especially by alleviating psychological distress in patients [11, 12]. The lack of improvement observed in the social domain may be attributed to the relatively short follow-up period of one month after discharge. Social participation generally requires a longer recovery time, greater physical capacity, higher self-confidence, and more comprehensive community support. Given that older adults typically experience slower rehabilitation, the focus during this early period is primarily on basic functional recovery, and most patients have not yet resumed active engagement in social activities. The distinct advantage of early weight-bearing training observed in the psychological domain appears to stem from its ability to effectively break the common “fear-avoidance” cycle in older adults with hip fractures. This effect is not solely due to physiological stimulation but is largely attributable to its strong psychological empowerment, which instills patients with a sense of hope and confidence in their own recovery. Psychological outcomes often respond more rapidly to such empowerment-based interventions compared with other domains.

In this study, Harris Hip Scores improved at all time points following the intervention in both groups, with more pronounced improvements observed in the intervention group. These findings indicate that early rapid rehabilitation exercises combined with early weight-bearing training can effectively promote hip function recovery after intertrochanteric femoral fractures. This is consistent with the results reported by He Yanqian [13], Ding Jian [14] and G. Song [15]. Early weight-bearing training can effectively prevent age-related bone loss and, when combined with exercises targeting core strength and balance, facilitates earlier restoration of walking ability [16]. Therefore, nurse should provide patients and their caregivers with training in early weight-bearing exercises following appropriate safety assessments, in order to enhance hip function and reduce caregiver burden.

The investigation revealed that adherence to rehabilitation exercises increased continuously in both groups of orthopedic patients, with a greater improvement observed in the intervention group. This indicates that early weight-bearing training is an effective strategy for enhancing patients’ adherence to rehabilitation exercises. It serves not only as a means to promote functional recovery but also as a behavioral intervention to optimize adherence. The underlying reasons for this may be twofold. First, early weight-bearing training in this study helped patients establish a cycle of “exercise execution – functional gain – motivation reinforcement.” Second, by setting a concrete goal of “restoring walking ability,” patients could directly perceive the progress of their daily function through daily weight-bearing exercises, which significantly enhanced their confidence in rehabilitation. These findings are consistent with those of Zhang Yiqun et al. [17], suggesting that multidisciplinary team guidance, through strengthened communication and encouragement, can further improve patient compliance. A recent international study offers important clues in this regard. Sağlam et al. [18] reported that adherence to standardized physiotherapy following the initial hip fracture was an independent predictor of the risk of sustaining a subsequent contralateral hip fracture. This work elevates “rehabilitation intervention” to the level of a public health strategy for preventing secondary fractures. In parallel, the higher adherence observed in the intervention group of our study represents a key element in ensuring the quality and effectiveness of rehabilitation care. These findings suggest that our intervention may not only have accelerated recovery from the initial fracture but also potentially established long-term protective factors by fostering proactive and consistent rehabilitation behaviors, thereby reducing the risk of subsequent fragility fractures. This provides a novel and clinically meaningful perspective for future studies evaluating the long-term benefits of early rehabilitation.

Trauma and surgical stimuli induce the release of a large number of inflammatory factors, which are key contributors to postoperative pain and limb swelling following fractures [19]. In this study, one day before discharge, significant differences in both resting and activity-related pain were observed between the two groups. At one month after discharge, only resting pain remained significantly different. The intervention group showed continuous pain improvement across all three time points, overall, the research results show that although no significant differences were observed in activity-related pain, the intervention may help relieve activity-related pain early before discharge, which requires further exploration. These findings indicate that early weight-bearing training may provide earlier and more sustained pain relief. The study by Bi Yuqi et al. [11] found that 66% of patients with hip fractures demonstrated insufficient postoperative rehabilitation due to fear of pain. Similarly, Lin Guandong et al. [20] investigated the application of the enhanced recovery after surgery (ERAS) protocol in older adults with hip fractures and reported that ERAS significantly reduced postoperative pain. This study demonstrated that standardized early weight-bearing training, implemented following safety assessments, was associated with earlier onset and more sustained postoperative pain relief compared with conventional rehabilitation. These results are consistent with the core tenets of ERAS frameworks in contemporary hip fracture management. The NICE guidelines [21] clearly recommend that for people with lower limb injuries, targeted weight-bearing exercise programs should be initiated. The aim is to enhance patients’ functions, such as mobility, the ability to move from sitting to standing, and lateral walking, through weight-bearing tasks, and to optimize pain control and functional recovery. A systematic review demonstrated that ultra-early rehabilitation with weight-bearing significantly alleviates postoperative pain in patients with hip fractures [22]. Our findings not only reinforce this international consensus, but more importantly, they provide an operational and reproducible approach for safely implementing early weight-bearing by quantifying load using a digital scale and establishing a defined pain-tolerance threshold (NRS ≤ 3). Therefore, clinicians and nursing staff may draw on such international evidence to more confidently communicate to patients that early weight-bearing under close monitoring is a safe and widely recommended strategy for postoperative analgesia and functional recovery, nurse should inform patients and their caregivers that appropriately designed early weight-bearing rehabilitation programs can play an important role in pain relief [23].

In this study, the intervention group should greater improvements exhibited in the motor subscale and total score of the Functional Independence Measure (FIM), indicating that early weight-bearing training may contribute to enhance functional independence following intertrochanteric femoral fractures. Early weight-bearing is thought to stimulates lower limb muscle contraction, improves blood circulation, and facilitates the clearance of inflammatory metabolites, potentially promoting functional recovery and enhancing mobility, including walking and transfer abilities [24]. On the other hand, weight-bearing activities might activate proprioceptors around the hip, restore balance, improve gait stability, and enhance overall mobility [25]. Notably, at one month after discharge, the duration of weight-bearing on the affected limb in the intervention group was significantly greater than that in the control group, challenging the traditional rehabilitation concept of “delayed weight-bearing to protect internal fixation.” The observed benefits of early weight-bearing could be attributed to mechanical stimulate that promotes osteogenesis, reduce pain sensitization, and enhances lymphatic return to alleviate swelling. Under conditions of stable internal fixation, absence of severe complications, and gradual progressive loading, and progressive loading, early weight-bearing appears to be a feasible, cost-effective, and potentially efficient component of postoperative rehabilitation. However, these findings require confirmation in larger, multicenter trials [24, 25].

## Conclusion

This study preliminarily demonstrates that, for older adults with intertrochanteric femoral fractures undergoing PFNA fixation, the implementation of a standardized early weight-bearing training program conducted under close monitoring, can safely promote hip function recovery, alleviate pain, and enhance both rehabilitation confidence and exercise adherence during the early postoperative period (within one month). These short-term positive outcomes align with the principles of international ERAS protocols and the recommendations of major clinical guidelines, providing a practical and actionable strategy to help achieve guideline-directed goals. However, these findings should be interpreted with caution. Limited by a one-month follow-up period and a moderate sample size, this study primarily assessed the short-term safety and efficacy of the intervention. In summary, as a promising early rehabilitation strategy, this intervention warrants further validation and exploration for broader application in clinical practice.

### Limitations

The present study has limitations. First, although early weight-bearing is widely recommended, the control group reflected prevailing local practice rather than current best evidence; thus, the findings compare a standardized early weight-bearing protocol with real-world delayed weight-bearing rather than early versus no weight-bearing. Second, Detailed intraoperative variables were not consistently collected, potentially resulting in residual confounding. Moreover, fracture stability classification and radiographic outcomes were not included. Prospective studies with standardized operative and imaging data are needed, our findings should not be interpreted as evidence of long-term implant stability or definitive functional recovery. Third, the follow-up period was relatively short and modest sample size, long-term outcomes remain unknown. Finally, post-discharge weight-bearing duration was caregiver-reported and may be subject to recall bias; future studies using wearable sensors could improve measurement accuracy.

## Supplementary Information

Supplementary Material 1.

Supplementary Material 2.

## Data Availability

The datasets used and/or analysed during the current study are available from the corresponding author on reasonable request.

## Declarations

### Copyright Statement

An unauthorized version of the Chinese MMSE was used by the study team without permission, however this has now been rectified with PAR. The MMSE is a copyrighted instrument and may not be used or reproduced in whole or in part, in any form or language, or by any means without written permission of PAR (www.parinc.com).

## Abbreviations

MMSE: Mini-Mental State Examination
FIM: Functional Independence Measure
PFNA: Proximal Femoral Nail Antirotation
ERAS: Enhanced Recovery After Surgery
NRS: Numeric Rating Scale
WHOQOL-BREF: World Health Organization Quality of Life Assessment Brief Form
HHS: Harris Hip Score
