# Supplementary material for: Effect of standardized early weight-bearing training on postoperative rehabilitation in older adults with intertrochanteric femoral fractures: a randomized controlled trial
Source: BMC Geriatr. 2026 Apr 22;26:571. doi: 10.1186/s12877-026-07533-4 (PMC13104271; doi:10.1186/s12877-026-07533-4)
Supplement: Supplementary file 1 — Supplementary Material 1. [file 12877_2026_7533_MOESM1_ESM.docx]

Supplementary Table 1. Linear Mixed-Effects Model Results for Harris Hip Score Over Time

| Fixed Effect | Estimate (β) | SE | 95% CI | F (df₁, df₂) | P-value |
| --- | --- | --- | --- | --- | --- |
| Intercept | 16.82 | 2.31 | [12.26, 21.38] | 130.71 | <0.001 |
| Group (Intervention) | –3.12 | 2.47 | [–7.99, 1.75] | 1.59 | 0.209 |
| Time (per unit increase) | 24.86 | 1.05 | [22.79, 26.93] | 567.26 | <0.001 |
| Group × Time | 6.34 | 1.75 | [2.88, 9.80] | 13.05 | <0.001 |

Supplementary Table 2. Parameter Estimates from Generalized Estimating Equation (GEE) Model for Repeated Measures of Resting Pain Score (N = 54)

| Predictor | β Coefficient | SE | 95% CI | P-value |
| --- | --- | --- | --- | --- |
| Group (Intervention vs. Control) | –0.185 | 0.075 | [–0.332, –0.039] | 0.013 |
| Time (vs. Follow-up) |  |  |  |  |
| Admission | 2.963 | 0.219 | [2.534, 3.392] | <0.001 |
| Pre-discharge | 0.963 | 0.133 | [0.702, 1.224] | <0.001 |
| Group × Time Interaction |  |  |  |  |
| Intervention × Admission | –0.148 | 0.339 | [–0.812, 0.516] | 0.662 |
| Intervention × Pre-discharge | –0.519 | 0.215 | [–0.940, –0.097] | 0.016 |

Supplementary Table 3. Parameter Estimates from Generalized Estimating Equation (GEE) Model for Repeated Measures of Activity-related Pain Score (N = 54)

| Predictor | β Coefficient | SE | 95% CI | P-value |
| --- | --- | --- | --- | --- |
| Group (Intervention vs. Control) | –0.148 | 0.178 | [–0.496, 0.200] | 0.404 |
| Time (vs. Follow-up) |  |  |  |  |
| Admission | 4.778 | 0.341 | [4.110, 5.446] | <0.001 |
| Pre-discharge | 2.407 | 0.198 | [2.020, 2.795] | <0.001 |
| Group × Time Interaction |  |  |  |  |
| Intervention × Admission | 0.407 | 0.411 | [–0.398, 1.212] | 0.321 |
| Intervention × Pre-discharge | –0.704 | 0.365 | [–1.419, –0.012] | 0.054 |
