# Supplementary material for: Effect of standardized early weight-bearing training on postoperative rehabilitation in older adults with intertrochanteric femoral fractures: a randomized controlled trial
Source: BMC Geriatr. 2026 Apr 22;26:571. doi: 10.1186/s12877-026-07533-4 (PMC13104271; doi:10.1186/s12877-026-07533-4)
Supplement: Supplementary file 2 — Supplementary Material 2. [file 12877_2026_7533_MOESM2_ESM.pdf]

April 14, 2026

Yue Ma
Huashan Hospital, Fudan University
12 Middle Urumqi Road
Shanghai, 200040
China

RE: Research titled, *Effect of standardized early weight-bearing training on postoperative rehabilitation in older adults with intertrochanteric femoral fractures: a randomized controlled trial.*

Dear Yue Ma:

This letter is to confirm receipt of payment for your unauthorized use of an unauthorized version of the Chinese MMSE in the research study referenced above (Invoice IN-00580041).

Also by way of this letter, you may use the data obtained from said research to publish the results of this study, subject to the following conditions:

1) The publication **must not include** any test items from the unauthorized version of the Chinese MMSE that was used without permission, or any other version of the MMSE in any language.

2) The publication **must include** this statement:
   An unauthorized version of the Chinese MMSE was used by the study team without permission, however this has now been rectified with PAR.
   The MMSE is a copyrighted instrument and may not be used or reproduced in whole or in part, in any form or language, or by any means without written permission of PAR (www.parinc.com).

3) One (1) copy of the article must be sent via email to PAR to confirm that the above conditions have been met.
   Email: afernandez@parinc.com

If you have any questions or concerns, please do not hesitate to contact me directly.

Sincerely,

Andrea Butler Fernandez
Jr. Permissions Specialist
afernandez@parinc.com
1-800-331-8378 (phone)
1-800-727-9329 (fax)
